# Hay Fever

**Published:** 1885-07

**Authors:** 


Hay Fever. By Chas. E. Sajous, M.D. Philadelphia, F. A. Davis, Att'y, 1885.

A little more than 200 pages are here devoted to the pathology, symptoms, and treatment of a disease which has hitherto puzzled, not a little, the profession, and brought discomfort and suffering, without hope of relief, to many unfortunates. The author has had great success in the treatment, which consists in a superficial organic alteration of the nasal mucous membrane by the application of an escharotic.

We have no doubt the book will be of great service to the profession.

The Oleates. By John V. Shoemaker, A.M., M.D. Philadelphia, F. A. Davis, Att'y, 1885.

This little book is invaluable to those who have occasion to treat cutaneous diseases. They will acknowledge, on reading it, that the oleates are destined to hold an important place in the therapeutics of the skin.

Its size permits it to be carried in the pocket, and its price ($1.00) brings it within the reach of all.

